# Extracts of *Spiraea hypericifolia* L. and *Spiraea crenata* L.: The Phenolic Profile and Biological Activities

**DOI:** 10.3390/plants11202728

**Published:** 2022-10-15

**Authors:** Olga A. Kaidash, Vera A. Kostikova, Elena V. Udut, Vladimir V. Shaykin, Denis R. Kashapov

**Affiliations:** 1 Central Research Laboratory, Siberian State Medical University, 634050 Tomsk, Russia; 2 Central Siberian Botanical Garden, Siberian Branch of Russian Academy of Sciences, 630090 Novosibirsk, Russia

**Keywords:** *Spiraea*, cytotoxicity, plant extract, antioxidant activity, phenolic compound, flavonoid

## Abstract

The comparative phytochemical analysis in this study revealed differences in the type and levels of phenolic compounds between *Spiraea hypericifolia* L. and *Spiraea crenata* L. The compounds in water–ethanol extracts of aerial parts of both species were identified by high-performance liquid chromatography as chlorogenic, gentisic, and cinnamic acids; quercetin; kaempferol; hyperoside; isoquercetin; nicotiflorin; and apigenin. In the extract of *S. hypericifolia*, *p*-coumaric acid and luteolin were also found, which were absent in the extract of *S. crenata*. Such compounds as avicularin, astragalin, and isorhamnetin-3-rutinoside proved to be specific to *S. crenata* (and were not found in the *S. hypericifolia* extract). The viability of liver cancer HepG2 cells and breast cancer MDA-MB-231 cells significantly decreased after cultivation with the *S. crenata* extract. In addition, the *S. crenata* extract showed higher antioxidant activity than the *S. hypericifolia* extract. It is most likely that these effects can be explained by the higher content of individual flavonoids in the extract of *S. crenata*. Thus, the extract of *S. crenata* holds promise for more extensive research on the mechanism of its action on tumor cells.

## 1. Introduction

Cancer is considered a leading cause of death and an important barrier to increasing life expectancy in all countries across the globe [1]. In 2020, there were 19.3 million new cases and 10 million deaths from cancer worldwide. Lung cancer in men and breast cancer in women are the most common malignant tumors and leading causes of cancer deaths [2]. Currently, most cancer patients undergo combined and complex treatments, including surgical operations, chemotherapy, and radiation therapy. Pharmacotherapy is under active development as new anticancer drugs come onto the scene: e.g., targeted drugs and immunotherapeutics [3,4]. For cancer treatment, the most popular drugs are cytostatics, but they have a number of serious adverse effects, such as immunosuppression, thrombocytopenia, and neutropenia. In this regard, the use of drugs as an adjunctive therapy in various schemes of combined and comprehensive treatments is becoming more relevant, because they raise the effectiveness of the cancer treatment. Acting as natural modifiers of biochemical reactions, herbal medicines are promising adjunctive therapeutics. The healing properties of medicinal plants are due to a wide range of pharmacologically active ingredients: alkaloids, glycosides, saponins, and other compounds [5,6].

Plants of the genus *Spiraea* L. are shrubs, and occur widely in Eurasia and North America. They are used in folk medicine as a diuretic, detoxifier, and pain reliever and to treat coughs, colds, inflammation, headaches, and toothaches [7,8,9,10]. The biological activity of the *Spiraea* species has been studied extensively, and to date, the high activity of some species has been identified and scientifically proven. For example, in recent studies, the *Spiraea* biological activity associated with the presence of phenolcarboxylic acids was well researched, and antidiabetic, insecticidal, and fungicidal effects were shown, as were properties that regulate plant growth [11,12,13]. Antiviral, antibacterial, antioxidant, and antilipogenic activities of extracts from leaves and inflorescences have been documented [9,14,15,16]. Acylated flavonoids isolated from *Spiraea* plants exert an anti–α-amylase effect [17]. A method was developed for obtaining a dry extract from the shoots of *S. salicifolia* L., which has manifested anti-inflammatory, diuretic, and antioxidant properties [18]. Antitumor activity of certain substances isolated from *Spiraea* representatives has been revealed. In the bark, leaves, and inflorescences of *S. hypericifolia* L., researchers have found phenolic compounds in effective doses with high biological activity: flavonols, flavones, flavans, and phenolcarboxylic acids [19,20,21]. Flavans isolated from the aerial part of *S. hypericifolia* [aglycones (+)-catechin and (−)-epicatechin, their glycosides 7-α-L-rhamnopyranoside (+)-catechin, 7-β-D-xylopyranoside (+)-catechin, 7-α-L-arabinoside (+)-catechin and dimers of catechins: (-)-epicatechin-(+)-catechin, and (−)-epicatechin-(−)-epicatechin] have relatively low toxicity and exert an antitumor action both alone and in combination with radiation therapy, in experiments in vivo and in vitro. The maximum tolerated flavan doses are 60–100 mg/kg of animal weight. In rats with Pliss lymphosarcoma and mice with Sarcoma 180, treatment with polyflavans has a significant antitumor effect. Rhamnoside (+)-catechin, in combination with irradiation in rats with Pliss lymphosarcoma, enhances the inhibition of tumor growth by 30% [20]. Chinese scientists have reported the antitumor activity of diterpene alkaloids from *S. japonica* L. A derivative of spiramine (with a β-unsaturated ketone group) isolated from *Spiraea* species is a new antitumor agent capable of inducing apoptosis of cancer cells [22,23].

*S. hypericifolia* and *S. crenata* L. are two of the most widespread species of *Spiraea* in nature and serve as abundant raw materials in Europe, the Caucasus, Central Asia, and Russia. *S. hypericifolia* also grows in Mongolia and China [24,25]. *S. crenata* has white flowers, 6–7 mm in diameter, and fluffy pedicels in numerous dense corymbs at the tops of short leafy twigs. In contrast to *S. crenata*, the flowers of *S. hypericifolia* are clustered in numerous few-flowered sessile umbels [24,25]. The extensive resource base and diverse pharmacological activities of representatives of the genus *Spiraea* suggest that *S. hypericifolia* and *S. crenata* are potential sources of biologically active substances with varying activities. Because the main active ingredients in *Spiraea* members are flavonoids and phenolcarboxylic acids, [16,17,26], our work aimed to determine the levels of phenolic compounds in water–ethanol extracts of *S. hypericifolia* and *S. crenata* and assess the antioxidant and cytotoxic properties of these extracts.

## 2. Results and Discussion

### 2.1. Phytochemical Analysis

The concentrations of phenolic compounds were determined in extracts from the two *Spiraea* species (Table 1). It was revealed that the total content of phenolic compounds and tannins is somewhat higher in the extract of *S. hypericifolia* (105.5 and 303.1 mg/g, respectively). The concentrations of phenolcarboxylic acids and catechins are also almost two times higher in *S. hypericifolia*, while the contents of flavonoids are higher in *S. crenata* (78.3 mg/g).

The profile and contents of phenolic compounds were analyzed by HPLC in the thick extracts from the plants (Table 2). Extracts from the *Spiraea* species studied share some compounds in common, but substances specific to each species were also identified. In the extracts of the two studied *Spiraea* species, common compounds are phenolcarboxylic acids (chlorogenic, gentisic, and cinnamic), aglycone flavonols (quercetin and kaempferol), quercetin glycosides (hyperoside and isoquercitrin), a kaempferol glycoside (nicotiflorin), and a flavone aglycone (apigenin). The main substances in the extract of *S. hypericifolia* are quercetin (6.6 mg/g), hyperoside (1.77 mg/g), apigenin (1.52 mg/g), luteolin, and kaempferol (1.09 mg/g each), whereas in the extract of *S. crenata,* isoquercitrin (2.48 mg/g), quercetin (2.40 mg/g), kaempferol (1.45 mg/g), apigenin (1.42 mg/g), and isorhamnetin-3-rutinoside (1.24 mg/g) were dominant. In the two extracts, the major compounds are either free or bound flavonoids. The main compounds in common between the two extracts are quercetin, kaempferol, and apigenin.

In the extract of *S. hypericifolia, p*-coumaric acid and luteolin were also found, which were not detectable in the *S. crenata* extract. Additionally, we identified quercetin glycoside (avicularin), kaempferol glycoside (astragalin), and isorhamnetin glycoside (isorhamnetin-3-rutinoside) in *S. crenata* samples, which were not found in *S. hypericifolia* samples.

According to the literature, antitumor activity has been detected in certain compounds in extracts from *S. hypericifolia* and *S. crenata.* Quercetin promotes cell cycle arrest and inhibits the growth of several cancer cell lines in vitro, such as leukemia cells and colon, breast, and ovarian cancer cells. Apigenin and luteolin induce apoptosis in several ovarian cancer cell lines (A2780, OVCAR-3, and SKOV-3) [27]. These data suggest a cytotoxic effect of the obtained extracts on various cell lines.

### 2.2. Cytotoxicity and Antioxidant Activity

Chinese scientists have revealed cytotoxic activity in an ethanol extract from *S. alpina* Pall; the extract has an inhibitory effect on the human liver BEL-7402 cancer cell line, colon cancer HCT-8 cells, and lung carcinoma A-549 cells [28].

Our cytotoxicity assay showed that the water–ethanol extract from the aerial part of *S. crenata* dose-dependently suppresses the viability of all the tested cell lines. The water-ethanol extract of *S. crenata* has the most significant cytotoxic effect against the human breast tumor MDA-MB-231 cell line. The half-maximal inhibitory concentration (IC_50_) is presented in Table 3.

The water-ethanol extract of *S. hypericifolia* did not exert any cytotoxic activity against the tumor cell line HepG2 and it disrupted the proliferation of 50% of the cells of the tested cell lines 3T3-L1 and MDA-MB-231 at higher concentrations than the water-ethanol extract from *S. crenata*. This means that there is a weaker cytotoxic effect for the water-ethanol extract of *S. hypericifolia*.

Examination of the cytotoxic activity of the extracts from aerial parts of *S. hypericifolia* and *S. crenata* was carried out for the first time. It is likely that the higher cytotoxic activity of the water-ethanol extract of *S. crenata* is explained by higher levels of certain flavonoids. Flavonoids are known to have many properties, such as anti-inflammatory, antitumor, cardioprotective, antimicrobial, and antiviral [29,30]. In our extract of *S. crenata*, the isoquercitrin content is almost four times higher than in the *S. hypericifolia* extract. One report suggests that isoquercetin dose-dependently reduces the viability of MDA-MB-231 and HepG2 cells by inducing apoptosis and autophagy through AMPK activation and inhibition of mTOR–p70S6K signaling in cells [31]. Here, avicularin, astragalin, and isorhamnetin-3-rutinoside were found in *S. crenata* samples, but not in *S. hypericifolia* samples. Isorhamnetin-3-rutinoside, the main component of the ethanol fraction of *Salsola oppositifolia* Desf. (*Amaranthaceae*), exerts a strong cytotoxic action against hormone-dependent human prostate adenocarcinoma LNCaP cells and invasive human ductal adenocarcinoma MCF-7 cells [32]. This flavonoid is one of the major components in our samples of the *S. crenata* extract. Another study has revealed that avicularin suppresses the viability of SCC13 skin squamous cell carcinoma cultured cells, by inducing apoptosis and inhibiting epithelial–mesenchymal transition [33]. It has been proposed that the mechanism of these effects against SCC13 cells is the modulation of genes related to apoptosis and epithelial–mesenchymal transition, as well as inhibition of the MEK–NF-κB signaling pathway.

**Figure 1:**
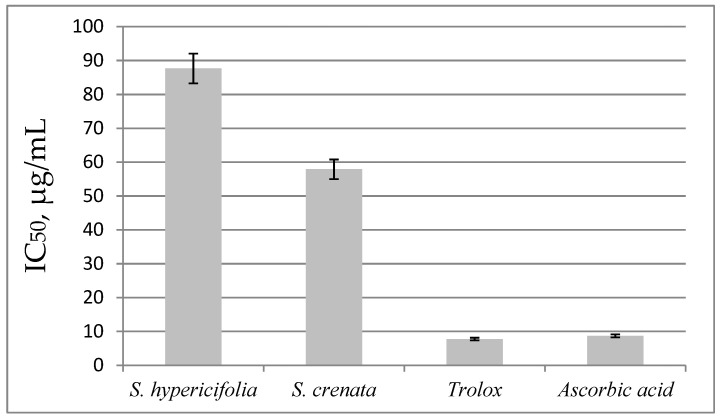
DPPH radical–scavenging activity of the two extracts. IC_50_, μg/mL of dry extract: concentration of an antioxidant at which 50% inhibition of the DPPH radical is observed.

Among the effects of flavonoids mentioned above, the most important one is the antioxidant effect, which differs depending on the type of functional group and its position in the compound’s molecule [34]. The high antioxidant potential of extracts from *Spiraea* plants has been repeatedly confirmed and often correlates with a high level of polyphenols. The methanol extract of *S. fritschiana* Schneid. has an antioxidant activity with an RC_50_ of 76.61 µg/mL [35]. The antiradical property of this extract is approximately 14 times lower than in the control (ascorbic acid, RC_50_ = 5.37 µg/mL). After they measured the reducing ability of the *Spiraea* plant extract at concentrations of 25–500 μg/mL, it was confirmed that the absorbance value (0.13–1.80) increased significantly with increasing extract concentration. In that study, the extract showed an approximately 50% reducing power compared to butylated hydroxytoluene (absorbance = 0.77), serving as a control at 100 μg/mL. Considering that only the crude extract from the aerial part of the *Spiraea* species was tested then, it can be said that its antiradical activity is relatively high. Furthermore, a high total level of phenolic compounds (212.78 μg/mg) and flavonols (66.84 μg/mg) is present in the extract [35]. The extract from *S. prunifolia* Sieb. et Zucc. *var. simpliciflora* Nakai has a high activity, similar to that of superoxide dismutase and high total levels of phenolic compounds (56.7 µg/mg) and flavonols (15.1 µg/mg) [36]. In another work, a methanolic extract of *S. prunifolia* var. *simpliciflora* manifested potent 1,1-diphenyl-2-picrylhydrazyl (DPPH) radical-scavenging activity and suppressed reactive oxygen species (ROS) production in NCI-H292 cells stimulated with tumor necrosis factor. This extract was effective at attenuating lipid peroxidation and restoring glutathione concentrations in lung tissues, in both a mouse model of lipopolysaccharide-induced acute lung injury and NCI-H292 cells stimulated with tumor necrosis factor [37]. In our assays, the DPPH method was employed to evaluate the antioxidant activity of the *Spiraea* extracts (Figure 1). The IC_50_ values were found to be lower for the extract of *S. crenata* (IC_50_ = 55.18 µg/mL). This extract inhibited the radical at lower concentrations than the extract of *S. hypericifolia* (IC_50_ = 87.67 µg/mL). The antioxidant activity of our standard substances (6-hydroxy-2,5,7,8-tetramethylchroman-2-carboxylic acid [Trolox] and ascorbic acid) was significantly higher than that of the tested *Spiraea* species’ extracts.

This finding of a much stronger DPPH radical suppression by the *S. crenata* extract is consistent with the observed toxicity to the analyzed cell lines and the higher levels of individual flavonoids. A number of authors have demonstrated the ability of various flavonoids to inhibit the cell cycle of tumor cells by influencing the production of ROS. For instance, genistein contributes to cell cycle arrest at the G2–M transition with subsequent ROS-dependent apoptosis, whereas naringenin lowers ROS production and enhances the activities of superoxide dismutase, catalase, peroxidase, and glutathione in tumor cells [38,39,40].

## 3. Materials and Methods

### 3.1. Plant Material

The material was collected in 2020 in natural populations (*S. hypericifolia*: in Russia, Novosibirsk Oblast, Toguchinsky district, vicinity of settlement Gorny, Mount Lysaya, steppe, N 54°54′, E 83°39′, 352 m a.s.l.; *S. crenata*: in Russia, the Republic of Altai, the left bank of the Koksa River, vicinity of village Ust-Koksa, south-east slope, open area, steppe forb meadow, N 50°17.994′ E 85°34.324′, 1021 m a.s.l.). Annual branches of plants were used for extraction: in *S. hypericifolia,* whole branches with leaves and flowers, and in *S. crenata,* whole branches too; the weight ratio of flowers, leaves, and stems in the latter case was 1.0:1.5:1.5. Air-dried plant material was crushed in an A11 basic mill (IKA, Staufen im Breisgau, Germany) and sifted through a 5 mm sieve.

### 3.2. Extract Preparation

The liquid extract was obtained in glass diffusers by the method of a three-stage countercurrent repercolation with a complete cycle, with a ratio of raw materials to the finished product of 1:1 [41]. A 40% ethyl ethanol solution was used as an extractant. The resulting liquid extract was dried in a dry evaporation vacuum system (Labconco, RapidVap, Kansas City, MO, USA) at 35 °C under pressure not exceeding 50 mBar. The residual moisture content of the obtained extracts was 2.9–3.8%.

To study the antioxidant activity and the biologically active substances contained in the extracts from the aerial parts of the two *Spiraea* species, 0.1 g of the thick extract must be dissolved in 10 mL of 40% aqueous ethanol.

### 3.3. Quantification of Phenolic Compounds

The total level of phenolic compounds was determined using the Folin–Ciocalteu reagent [42]. An extract (0.5 mL) was placed in a 5-mL volumetric flask, then 2.5 mL of the Folin–Ciocalteu reagent (diluted at 1:10 with distilled water) and 2 mL of a 7.5% aqueous sodium carbonate solution were added and shaken well. The mixture was kept at 45 °C in a water bath for 15 min. Absorption was measured at a wavelength of 765 nm on an SF-56 spectrophotometer (Lomo, St. Petersburg, Russia). A blank sample consisting of distilled water and reagents served as a control. The standard curve was constructed with gallic acid (concentration 0.002–0.01 mg/mL), and the total phenolic content was expressed in milligrams of gallic acid equivalents per gram of a dry extract by means of the standard curve.

### 3.4. Determination of Flavonol Contents

This procedure was performed by a spectrophotometric method based on the complex formation reaction between flavonols and aluminum chloride [43]. An extract (0.1 mL) was placed into two 5 mL test tubes, 0.2 mL of a 2% ethanol solution of aluminum chloride was added into one test tube, 1–2 drops of 30% acetic acid were added into the other, and the solution was brought to the nominal volume with 96% ethanol. The solutions were mixed, and after 40 min, the optical density of the solution with aluminum chloride was measured on the SF-56 spectrophotometer (Lomo, St. Petersburg, Russia) at a wavelength of 415 nm in a cuvette with a light path of 1 cm, using a solution of acetic acid as a control. The amount of flavonols in each sample was determined by a calibration curve built based on rutine (Chemapol, Mumbai, MH, India), concentration 0.01–0.1 mg/mL. The results were expressed in milligrams of rutine equivalents per gram of dry extract using the standard curve.

### 3.5. Quantitation of Catechins

The concentration of catechins was determined spectrophotometrically by the method based on the ability of catechins to produce a crimson color in a solution of vanillin in concentrated hydrochloric acid [44,45]. A 0.8 mL aliquot of extract was placed into two test tubes. Next, 4 mL of a 1% solution of vanillin in concentrated hydrochloric acid was poured into one of them, and the volumes were adjusted to 5 mL in both tubes with concentrated hydrochloric acid. A tube without vanillin was utilized as a control. If catechins were present, the sample turned pink, raspberry, or orange-red. After 5 min, the intensity of colors was measured on SF-56 (Lomo, St. Petersburg, Russia) at 504 nm in a cuvette with a light path of 1 cm. The standard curve was constructed with (±)-catechin (Sigma, St. Louis, MO, USA), concentration 0.001–0.02 mg/mL. The content of catechins was expressed in milligrams of (±)-catechin equivalents per gram of a dry extract using the standard curve.

### 3.6. Quantification of Tannins

Quantitation of tannins (hydrolyzable tannins) was performed by the method proposed by L.M. Fedoseeva [46]. An extract (10 mL) was transferred into a 100 mL volumetric flask, and 10 mL of a 2% aqueous solution of ammonium molybdate was introduced. The flask contents were brought to the nominal volume with purified water and incubated for 15 min. The intensity of the resulting color was measured on the SF-56 spectrophotometer (Lomo, St. Petersburg, Russia) at 420 nm in a cuvette with a light path of 1 cm. A government standard sample of tannin (Sigma, St. Louis, MO, USA, concentration 0.01–0.1 mg/mL) served as a standard. The results were expressed in milligrams of tannin equivalents per gram of dry extract using the standard curve.

### 3.7. Quantitation of Total Phenolic Acids

The total level of phenolic acids was determined using Arnov’s reagent [47,48]. To 1 mL of extract, we added 5 mL of distilled water, 1 mL of hydrochloric acid (0.1 mol), 1 mL of Arnov’s reagent (10.0 g of sodium molybdate and 10.0 g of sodium nitrate in 100.0 mL of water), 1 mL of sodium hydroxide (1 mol), adjusted to 10 mL with distilled water, and the optical density was measured immediately at 490 nm on the SF-56 spectrophotometer (Lomo, St. Petersburg, Russia). A blank sample consisting of distilled water and reagents was used as a control. Results were expressed in milligrams of caffeic acid (Serva, Heidelberg, Germany, concentration 0.02–0.1 mg/mL) equivalents per gram of dry extract, using the standard curve.

### 3.8. HPLC Assays of the Profile and Levels of Phenolic Compounds in the Extracts

One milliliter of a water-ethanol extract was diluted with double-distilled water to 5 mL and passed through a Diapak C16 concentrating cartridge (BioKhimMak Co., Moscow, Russia). The substances were washed off the cartridge with a small amount (3 mL) of 40% ethanol and then with 2 mL of 96% ethanol. The combined eluate was passed through a membrane filter with a pore diameter of 0.45 μm.

The phenolic compounds in the eluate were analyzed on an analytical HPLC system consisting of an Agilent 1200 liquid chromatograph (USA) with a diode array detector, an autosampler, and a ChemStation system for collecting and processing of chromatographic data by the van Beek method [49] with modifications. A Zorbax SB-C18 column (4.6 × 150 mm, 5 µm) was employed. Chromatographic analysis was first carried out via isocratic elution in a methanol–0.1% orthophosphoric acid (31:69) mixture for 27 min, then in a gradient elution mode: in the mobile phase, the proportion of methanol in an aqueous solution of orthophosphoric acid (0.1%) was changed from 33% to 46% during 11 min, then from 46% to 56% during the next 12 min, and from 56% to 100% for 4 min at an eluent flow rate of 1 mL/min and column temperature 26 °C. The volume of the injected sample was 10 µL. Detection was conducted at wavelengths 254, 270, 290, 340, 360, and 370 nm.

The quantification of phenolic compounds was conducted as previously reported [50]. To prepare standard samples, gentisic and cinnamic acids (Serva Heidelberg, Germany), chlorogenic and *p*-coumaric acids, quercetin, kaempferol, luteolin, apigenin, nicotiflorin, isorhamnetin-3-rutinoside (Sigma-Aldrich, St. Louis, MO, USA) isoquercitrin, avicularin, astragalin, and hyperoside (Fluka, Everett, Washington) were employed. Standard solutions were prepared at a concentration of 10 µg/mL.

### 3.9. The Cytotoxicity Assay

The cell line of noncancerous mouse fibroblasts (3T3-L1) and the epithelial liver cancer cell line HepG2 were both acquired from Vector (Novosibirsk, Russia), and human mammary gland cancer MDA-MB-231 cells from ECACC (Porton Down, UK). The cells were cultured in Dulbecco’s modified Eagle’s medium/F12 (Sigma-Aldrich, St. Louis, MO, USA) supplemented with 10% of fetal bovine serum (FBS), 1% of a GlutaMAX glutamic acid solution (Gibco, Massachusetts, USA), and 50 g/L of antibiotic (Gentamicin) in a humid atmosphere containing 5% of CO_2_ at 37 °C. To evaluate the cytotoxicity of the *S. hypericifolia* and *S. crenata* extracts, the 3-(4,5-methylthiazol-2-yl)-2,5-diphenyl tetrazolium bromide MTT assay was chosen, with a slight modification. The method is based on the ability of mitochondrial dehydrogenases in a live cell to reduce water-soluble MTT to formazan crystals. Briefly, after the passaging of cells in 96-well plates (SPL Life Sciences Co., Ltd., Pocheon, Korea) at 10,000 per well, the plates were kept in a CO_2_ incubator (Sanyo, Moriguchi, Japan) for 24 h for cell adhesion and to achieve 80–90% confluence. The next day, stock solutions of the extracts in DMSO were prepared, as were serial twofold dilutions. The concentrations of the samples were 1000–7.81 µg/mL. Then 1 μL of such a solution was added into the wells of a plate with cells, while the concentration of DMSO was 0.5%. The equivalent volume of DMSO was added to the control cells.

After the addition of the tested extracts, the plates with cells were kept in a CO_2_ incubator for 24 h. After that, the medium in the wells was replaced with a fresh one, and 10 μL of the MTT reagent (5 mg/mL) dissolved in sterile PBS was introduced into each well. After 2 h of incubation, the medium was removed from the wells, and 200 µL of DMSO was added into each well to dissolve the formazan crystals. The absorbance of the resultant solutions was measured in each well at 540 nm and at a reference wavelength of 650 nm on an Infinite M Plex automated microplate analyzer with a Te-Inject system (Tecan Austria GmbH, Grödig, Austria). The concentration of the extract at which the viability of the cell culture was reduced to 50% of the control (IC_50_) was calculated.

### 3.10. The Antioxidant Activity Assay Using DPPH

The ability of the samples to scavenge free radicals was determined by the DPPH method [51,52]. For this purpose, a 2 mL aliquot of an extract (the extracts were diluted with 40% ethanol to concentrations in the range of 20–1200 µg/mL) was mixed with 3 mL of a DPPH solution (62 µg/mL in ethanol). After 40 min incubation in the dark at room temperature, absorbance (D) was measured at 517 nm against a blank. Free-radical–scavenging activity was calculated as a percentage inhibition via the following formula:

X% = (D_control_ − D_sample_/D_control_) × 100, (1)

where D_control_ is the optical density of the control solution containing all reagents except for the tested extract, and D_sample_ is the optical density of the sample.

The results are expressed in DPPH IC_50_, defined as the antioxidant concentration that causes a 50% loss of DPPH in the DPPH radical-scavenging activity assay. Solutions of Trolox and ascorbic acid (concentrations 2.5–50.0 μg/mL) served as a positive control.

### 3.11. Statistical Analysis

This analysis was carried out in GraphPad Prism v8.4.3 and Microsoft Excel 2016. The assays of antioxidant activity and of the levels of phenolic compounds were performed on three technical replicates. A Student’s *t*-test was performed to determine the statistical significance of the difference in substance levels between the extracts of *Spiraea* species.

## 4. Conclusions

The profile and contents of phenolic compounds in water-ethanol extracts from two *Spiraea* species that are widespread in nature—*S. hypericifolia* and *S. crenata*—were studied. Differences in the phenolic profile were revealed between the two *Spiraea* species analyzed. A significant decrease in the viability of liver cancer HepG2 cells and breast cancer cell line MDA-MB-231 was registered after culturing with the extract of *S. crenata*. In addition, this extract has a higher antioxidant activity. It is most likely that these effects are attributable to the concentrations of certain flavonoids (avicularin, astragalin, and isorhamnetin-3-rutinoside) in *S. crenata*.

## Figures and Tables

**Table 1:** Contents (mg/g of dry extract) of phenolic compounds in extracts from the two *Spiraea* species.

Species	Catechins	Phenolcarboxylic Acids	Flavonoids	Tannins	Phenolic Compounds
*S. hypericifolia*	6.12 ± 0.53 *	66.22 ± 2.04 *	69.74 ± 2.32 *	303.11 ± 27.28 *	105.51 ± 9.50 *
*S. crenata*	2.4 ± 0.22	37.30 ± 3.36	78.31 ± 1.61	220.23 ± 19.82	68.59 ± 6.17

The table shows the mean and standard deviation (*n* = 3). Statistically significant differences are marked with an asterisk (*) (Student’s *t*-test at *p* < 0.05).

**Table 2:** Characteristics and contents of phenolic compounds identified by HPLC in extracts from aerial parts of *S. hypericifolia* and *S. crenata*.

Peak Number	Compound (Spectral Characteristics, λ_max_, nm)	Retention Time, min	Content mg/g of Dry Extract	
			*S. hypericifolia*	*S. crenata*
Extracts				
1	Chlorogenic acid (244, 330)	3.2	0.30 ± 0.01 *	0.53 ± 0.01
2	Gentisic acid (235, 330)	4.7	0.39 ± 0.01 *	0.14 ± 0.01
3	*p*-Coumaric acid (226, 310)	7.9	0.27 ± 0.01	–
4	Hyperoside (255, 355)	18.0	1.77 ± 0.02 *	0.57 ± 0.01
5	Isoquercitrin (259, 358)	19.3	0.65 ± 0.01 *	2.48 ± 0.03
6	Avicularin (260, 360)	28.4	–	0.43 ± 0.01
7	Astragalin (265, 350)	32.5	–	0.37 ± 0.01
8	Nicotiflorin (260, 350)	33.1	0.73 ± 0.01 *	0.41 ± 0.01
9	Cinnamic acid (216, 275)	35.0	0.67 ± 0.01 *	0.63 ± 0.01
10	Isorhamnetin-3-rutinoside (250, 350)	35.8	–	1.24 ± 0.01
11	Quercetin (255, 372)	40.6	6.62 ± 0.07 *	2.40 ± 0.03
12	Luteolin (255, 355)	44.0	1.09 ± 0.01	–
13	Kaempferol (260, 370)	47.5	1.09 ± 0.01 *	1.45 ± 0.02
14	Apigenin (270, 340)	49.6	1.52 ± 0.02 *	1.42 ± 0.02

The table shows the mean ± standard error (*n* = 3); “–”: a compound is absent. Statistically significant differences are marked with an asterisk (*) (Student’s *t*-test at *p* < 0.05).

**Table 3:** Cytotoxic activity of water-ethanol extracts from aerial parts of *S. hypericifolia* and *S. crenata*.

	IC_50_ toward Cell Lines, µg/mL of Dry Extract		
	3T3-L1	HepG2	MDA-MB-231
*S. hypericifolia*	886 ± 63	>1000	746 ± 23
*S. crenata*	291 ± 56	266 ± 13	124 ± 19

The table shows the mean ± standard error (*n* = 3). 3T3-L1: a cell line of noncancerous mouse fibroblasts, HepG2: a human liver epithelial cancer cell line, MDA-MB-231: a human breast cancer cell line.

## Data Availability

Not applicable.
